# Anti-tumor effects and mechanism of GA-13315, a novel gibberellin derivative, in human lung adenocarcinoma: an in vitro and in vivo study

**DOI:** 10.1186/s11658-018-0126-9

**Published:** 2019-01-10

**Authors:** Lin Xie, Yajuan Chen, Jingbo Chen, Hongbin Zhang, Yedan Liao, Yonghong Zhou, Ling Zhou, Chen Qing

**Affiliations:** 1 Department of Medical Oncology, Third Affiliated Hospital of Kunming Medical University/ Cancer Hospital of Yunnan Province, Kunming, China; 2 School of Pharmaceutical Science and Yunnan Key Laboratory of Pharmacology for Natural Products, Cancer Hospital of Yunnan Province, Kunming Medical University, NO.1168, West Chunrong Road, Chenggong Developing Area, Kunming, 650031 China; 3 Key Laboratory of Medicinal Chemistry for Natural Resource, Ministry of Education School of Chemical Science and Technology, Yunnan University, Kunming, China

**Keywords:** Gibberellin derivatives, Lung adenocarcinoma, Antitumor, Toxicity

## Abstract

**Objective:**

To investigate the anti-tumor effects and the mechanism of the compound 13-chlorine-3, 15-dioxy-gibberellic acid methyl ester (GA-13315) in lung adenocarcinoma in vitro and in vivo.

**Methods:**

The antiproliferative effect of GA-13313 on the A549 cell line was determined by MTT (3-[4, 5-dimethylthiazol-2-yl]-2, 5 diphenyl tetrazolium bromide) assay. A xenograft model of A549 was established to evaluate the anti-tumor effect and histopathological examination was performed to assess the toxicity of GA-13315. Apoptosis was detected by TUNEL staining in tissues and flow cytometry in cells; activation of caspase-3, caspase-8 and caspase-9 was evaluated by immunohistochemical analysis; protein levels of Bcl-2-associated X protein (Bax), B-cell lymphoma-2 (Bcl-2), caspase-4, activating transcription factor 4 (ATF4), glucose-regulated protein 78 (GRP78) and growth arrest and DNA damage-inducible gene 153 (GADD153) were determined by western blotting. Mitochondrial membrane potential (MMP) was measured by the JC-1 fluorescence probe.

**Results:**

Our results showed that GA-13315 exhibited potent, dose- and time-dependent anti-proliferative activity, and the IC50 values were 37.43 ± 2.73, 28.08 ± 7.76 and 19.29 ± 7.61 μM at 24, 48, and 72 h, respectively. The xenograft experiment revealed that tumor weight and volume were significantly decreased after GA-13315 3 mg/kg and 9 mg/kg (*P* < 0.05) treatment, and GA-13315 had low toxicity in bone marrow, kidney and colon tissues. GA-13315 triggered remarkable apoptosis in A549 cells at the concentration of 25.6 μM and 32 μM (*P* < 0.05) and activated caspase-3, − 8 and − 9. Moreover, GA-13315 induced apoptosis through the mitochondrial apoptosis pathway by elevating the Bax/Bcl-2 ratio, releasing cytochrome c and activating caspase-9 in A549 cells. In the endoplasmic reticulum apoptosis pathway, the levels of caspase-4, ATF4, GRP78 and GADD153 were markedly upregulated.

**Conclusions:**

This study suggests that GA-13315 can be considered as a promising chemotherapeutic agent with anticancer activity in treatment of lung cancer in future.

## Introduction

As one of the most common malignancies, lung cancer has been the leading cause of cancer-related mortality worldwide [1], with a reported death rate of 610.2/100,000 in China alone [2]. Non-small cell lung carcinoma (NSCLC) accounts for approximately 80% of all lung cancer victims, of which adenocarcinoma is the main subtype [3]. Most cases of lung adenocarcinoma are generally diagnosed with locally advanced or metastatic diseases [4, 5]. Currently, traditional chemotherapy with various chemotherapeutic agents such as cisplatin (DDP), paclitaxel, carboplatin, etc., even the targeted drugs epidermal growth factor receptor-tyrosine kinase inhibitors (EGFR-TKIs), has been established as the preferred anticancer therapy in clinical practices [6, 7]. However, the efficacy and survival in patients with lung adenocarcinoma remain limited by relapse, drug resistance and drug-induced toxicity [8, 9].

It is well known that natural products played a critical role in anticancer discovery due to their structural diversity [10]. Therefore, as a new strategy for anticancer therapy, the development of natural drug has attracted considerable interest. Gibberellin, a member of tetracyclic diterpenes, biosynthesized from entkaurenes, have shown strong antitumor bioactivities; however, the applications of gibberellins are still limited as plant growth regulators [11, 12]. GA-13315 is a novel synthetic gibberellin derivative, and possesses potent antitumor activity due to the existence of an α,β-unsaturated ketone moiety [13]. Our previous study demonstrated the inhibitory effect of GA-13315 on proliferation of A549 cells in xenograft mice models [12]. In addition, more recent evidence revealed that GA-13315 inhibited the growth and proliferation of oral, breast, and leukemia tumor cells through exerting antiangiogenic activity [12, 14], and the value of inhibitory concentration 50 (IC_50_) in various tumor cell lines ranged from 0.13 to 30.28 μg/ml. However, the antitumor effect and the underlying mechanism of GA-13315 on human lung adenocarcinoma have not been fully evaluated.

In the present study, we aimed to explore the antitumor effect of GA-13315 on human lung adenocarcinoma (A549 cells) in vitro and in vivo, as well as its apoptosis mechanism. Hopefully, the findings of the present study will provide new evidence on the development of a natural antitumor drug for lung adenocarcinoma.

## Materials and methods

### Cell line and culture

Non-small cell lung adenocarcinoma cell line A549 was purchased from Shanghai Institute of Materia Medica, Chinese Academy of Sciences (Shanghai, China). Cells were cultured in DMEM/F12 medium with 10% (*v*/v) fetal bovine serum (FBS) and incubated at 37 °C in an atmosphere of 5% CO_2_ and 95% air. GA-13315 was prepared by the School of Chemical Science and Technology, Yunnan University (Yunnan, China). The stock concentrations of GA-13315 (50 mg/ml) were prepared in dimethyl sulfoxide (DMSO) and stored at − 20 °C for the following tests.

### MTT assay

The effect of GA-13315 on cell proliferation was measured using the MTT assay. Briefly, A549 cells were seeded into 96-well plates with a density of 8 × 10^3^ cells/well. Then GA-13315 at a series of concentrations (4, 8, 16, 32, 64, and 128 μM) was added. After incubation for 24 h, 48 h and 72 h, 20 μL of MTT (Sigma Aldrich, US) from a stock solution (0.5 mg/mL) was added to each well, and incubated for an additional 4 h. The optical densities of the obtained formazan crystals were measured at 570 nm and 630 nm. 50% inhibitory concentration (IC_50_) values were calculated by the LOGIT model. Independent experiments were performed in triplicate.

### Apoptosis detection in A549 cells by TUNEL staining

After GA-13315 treatment for 48 h, the cells were centrifuged for 5 min at 1000 g (about 2000 rpm). Then the supernatant was discarded, and the cells were washed with phosphate-buffered saline (PBS) twice. After centrifugation, the cells were collected and fixed with 4% paraformaldehyde for 30–60 min. After washing with PBS twice, fixed cells were suspended and apoptotic cells were stained using terminal deoxynucleotidyl transferase dUTP nick-end labeling (TUNEL) assays with the In-Situ Cell Death Detection Kit (Roche, USA) according to the manufacturer’s instructions. Flow cytometry was performed to determine the apoptosis rates.

### Separation of mitochondria and cytoplasmic Cyt C

Forty-eight hours after GA-13315 treatment, A549 cells were collected by centrifugation at 1000 g (about 2000 rpm) for 5 min, then the supernatant was discarded, and the cells were collected, and then the cells were washed twice with PBS. After homogenization, the homogenate was centrifuged at 1000 g for 10 min at 4 °C; the supernatant was then transferred to another centrifuge tube, centrifuged at 11,000 g for 10 min at 4 °C, and the precipitate was mitochondria. The collected supernatant was then centrifuged at 12,000 g for 10 min at 4 °C, and the supernatant was cytoplasmic protein without mitochondria. RIPA cell lysate was used for further obtaining cytoplasmic Cyt C, followed by Western blot detection.

### Western blot analysis

For Western blot analysis, A549 cells were washed twice with cold PBS and then lysed on ice in PMSF lysis buffer at 4 °C for 30 min. The supernatant was collected after sonication and centrifugation, and the protein concentration was determined using the BCA protein assay. The protein homogenates were separated by 10% SDS-PAGE and transferred to polyvinylidene difluoride (PVDF) membranes. The membranes were blocked with 5% non-fat milk in NaCl/Tris-T (1 mM Tris/HCl, pH 6.8) buffer for 1 h and then incubated with primary antibodies (caspase-9, Bax and Bcl-2 from Santa Cruz, CA, USA) overnight at 4 °C. The membranes were incubated with labeled horseradish peroxidase (HRP) secondary antibodies goat anti-rabbit IgG (Santa Cruz, CA, USA) at room temperature for 1 h, and then visualized with the enhanced chemiluminescence (ECL) detection system (GE Healthcare, USA).

### Mitochondrial membrane potential assay

The mitochondrial membrane potential (MMP) was analyzed using the JC-1 fluorescence probe (5,5′,6,6′-tetrachloro-1,1′,3,3′-tetraethylbenzimidazolocarbocyanine iodide, Molecular Probe, USA). The fluorescent dye JC-1 is capable of selectively entering mitochondria, where it forms aggregates and emits red fluorescence when MMP is high, while at low MMP, JC-1 cannot enter mitochondria and forms monomers that emit green fluorescence. The ratio of red to green fluorescence is an indicator of cell apoptosis. The A549 cells were treated with JC-1 at 37 °C for 1 h. After washing with serum-free medium, the fluorescence intensity was immediately measured using a laser scanning confocal microscope (Carl Zeiss, Germany).

### Xenograft model and treatment

All experimental procedures in this study conform to the National Institutes of Health Guide for the Care, and Use of Laboratory Animals, and were approved by the Institutional Animal Care and Use Committee of Kunming Medical University. Male BALB/c nude mice (6–7 weeks, 20 ± 2 g) were obtained from Charles River Laboratory Animal CO., LTD. (Beijing, China) (No. SCXK 2012–0001). All mice were fed ad libitum, and were housed under a 12 h light/dark cycle at a temperature of 25 ± 2 °C and 60% humidity. A549 cells (7 × 10^7^/0.2 ml) were subcutaneously inoculated in nude mice. When tumor volumes reached approximately 1 × 1 × 1 cm^3^, subcutaneous tumors were collected and cut into 1.5 mm^3^ slices and then transplanted into subaxillary tissues of male BALB/c nude mice.

When the tumors reached a mean volume of 1–3 mm^3^, the mice were randomly assigned to five groups (*n* = 6): control group (0.5% CMC-Na, five times a week for 4 weeks); DDP group (1 mg/kg, five times a week for 4 weeks); GA-13315 (1 mg/kg group, 3 mg/kg group and 9 mg/kg) groups (five times a week for 4 weeks). The mice were intraperitoneally injected (i.p) with 0.5% CMC-Na in the negative control group and with DDP in the positive control group, while mice in treatment groups were gavage administered with GA-13315. Mice behavior and body weight of each mouse were measured every three days. The mice were sacrificed after the last measurement; tumors and important organs were removed and collected immediately. Tumor volume (mm^3^) was measured with calipers and calculated as (width^2^ × length)/2, and relative tumor volume (RTV) was also calculated. The tumor relative growth rate (T/C%) and the tumor growth inhibition rate were calculated as described previously (12).

### Determination of apoptosis by transmission electron microscopy

The morphological characteristics of tumor tissue were observed by transmission electron microscope. Tumor tissues (1 cm) were sectioned and fixed in 2.5% glutaraldehyde followed by rinsing in phosphate buffer (0.1 mmol/L) 3 times. The sections were post-fixed with osmium tetroxide (0.1 mmol/L), dehydrated with graded dilutions of ethanol and acetone, and then embedded in resin. Ultrathin sections (50 nm) were prepared and stained with uranyl acetate and lead acetate. Samples were detected using a transmission electron microscope (H-800, Hitachi Ltd., Tokyo, Japan).

### TUNEL assay and immunohistochemical analysis in tumor tissues

Tumor tissues were immediately resected from the animals at the time of sacrifice. The tissues were fixed with 4% paraformaldehyde and embedded in paraffin. 4 μm paraffin sections were then dewaxed and incubated with proteinase K (20 μg/mL dissolved in Tris/HCl, Ph 7.4–8.0) at 37 °C for 20 min. After washing with PBS twice, a mixed TUNEL solution of 50 μL was added and incubated for 60 min at 37 °C in a wet box. Then 50 μL of the conversion agent POD was added, and was incubated in the wet box for 30 min at 37 °C. Samples were next washed with PBS 3 times, and 100 μL of diaminobenzidine (DAB) was added following incubation for 20 min at room temperature. The staining results were analyzed under a light microscope.

Immunohistochemical (IHC) analysis for caspase-3, − 8 and − 9 was performed using 5 μm-thick paraffin-embedded tumor sections. The sections were dewaxed and rehydrated for 20 min. EDTA (pH 9.0) was utilized for antigen retrieval. Endogenous peroxidase activity was blocked with 3% H_2_O_2_ for 10 min at 37 °C, and then incubated with normal goat blood serum to block nonspecific binding sites. Then sections were incubated with primary antibodies (caspase-3, caspase-8, and caspase-9 from Santa Cruz, CA, USA) at 4 °C, and washed with PBS before incubation with secondary antibody (Santa Cruz, CA, USA). DAB was used as a chromogen, followed by counter-staining with hematoxylin, dehydrated and mounted.

Positive TUNEL staining was localized in the nucleus, and positive caspase-3, − 8 and − 9 staining was localized in the cytoplasm. To evaluate staining, a score corresponding to the sum of (a) the percentage of positive cells in each field of view: 0, 0–5%; 1, 6–25%; 2, 26–50%; 3, 51–75%; 4, >75% and (b) the staining intensity according to the staining characteristics of most positive cells (0, non-staining; 1, faint yellow; 2, clay bank; 3, sepia) was established. The sum of a plus b was considered as comprehensive scores: 0–1, negative; 2–3, weakly positive; 4–5, positive; 6–7, strongly positive. The results of staining were assessed by 2 independent pathologists in a blinded manner.

### Assessment of toxicity

The bone marrow, colon and kidney tissues were prepared for toxicity analysis using Wright’s and hematoxylin-eosin (HE) stain. Wright-Giemsa dye solution (Regen biotechnology co. LTD., Beijing, China) was applied to dry bone marrow smear, and then twice the amount of buffer solution was added and thoroughly mixed for 15–20 min. The number of nucleated cells in a bone marrow slice image is an important index of the degree of hyperplasia. The smears were visualized and the percentage of nucleated cells was calculated. Colon and kidney tissues were fixed in 10% formalin phosphate buffer and consequently processed for paraffin embedding. The 5 μm sections were prepared and stained with HE.

### Statistical analysis

Statistical analysis was performed using SPSS software (Version 19.0, SPSS Inc., Chicago, IL, USA). The measurement data are presented as mean ± standard deviation. Mean comparison in groups was conducted by one-way analyses of variance (ANOVA), and followed by Student’s t test. *P* < 0.05 was considered statistically significant. 

## Results

### Anti-proliferative activity of GA-13315 against A549 cells in vitro and in vivo

**Fig. 1:**
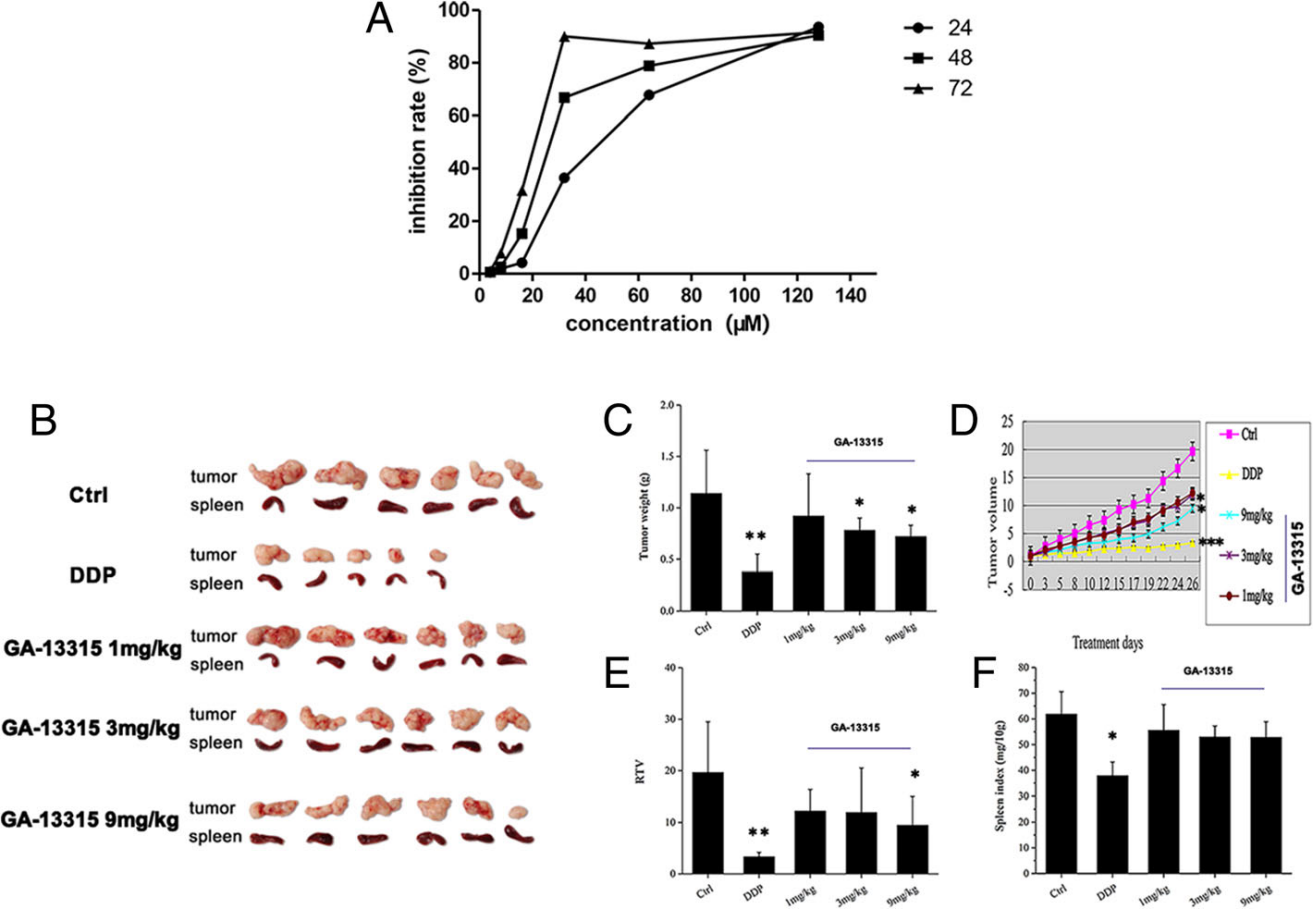
GA-13315 inhibition of human lung adenocarcinoma A549 cell proliferation. **a**, GA-13315 inhibited proliferation of A549 cells; **b**, Photographs of excised tumors and spleens; **c**, Tumor weight of xenograft model of A549 in control group and GA-13315 treated groups; **d**, Tumor volume of xenograft model of A549 in control group and GA-13315 treated groups; **e**, RTV of xenograft model of A549 in control group and GA-13315 treated groups; **f**, Spleen index of xenograft model of A549 cells in control group and GA-13315 treated groups. Ctrl, control group; DDP, cisplatin; RTV, relative tumor volume. *P < 0.05, compared with control group; **P < 0.01, compared with control group. ***P < 0.001, compared with control group

In this study, the anti-proliferative activity of GA-13315 against the human lung adenocarcinoma cell line A549 was assessed by MTT assay in vitro. The IC_50_ values at 24, 48, and 72 h were 37.43 ± 2.73 μM, 28.08 ± 7.76 μM and 19.29 ± 7.61 μM, respectively. Our results showed that GA-13315 exhibited potent, dose- and time-dependent anti-proliferative activity at 24, 48, and 72 h (Fig. 1A).

The anti-tumor efficacy of GA-13315 in vivo was further investigated in a xenograft model of the A549 cell line in nude mice. Photographs of representative tumors and spleens excised on day 26 are shown in Fig. 1B. As shown in Fig. 1C and D, the tumor weight and volume in the control group grew rapidly, and the tumor weight and volume were significantly decreased after treatment with GA-13315 3 mg/kg and 9 mg/kg. The RTV in the GA-13315 9 mg/kg group was markedly reduced (Fig. 1E). In addition, we also found that the T/C in GA-13315 treatment group was 61.07% (1 mg/kg), 59.77% (3 mg/kg) and 47.33% (9 mg/kg), respectively, with an obvious reduction compared with the control group. However, there was no significant difference in spleen index between the GA-13315 treatment group and control group (Fig. 1F).

### Toxicity evaluation of GA-13315 treatment – Histology of main organs

**Fig. 2:**
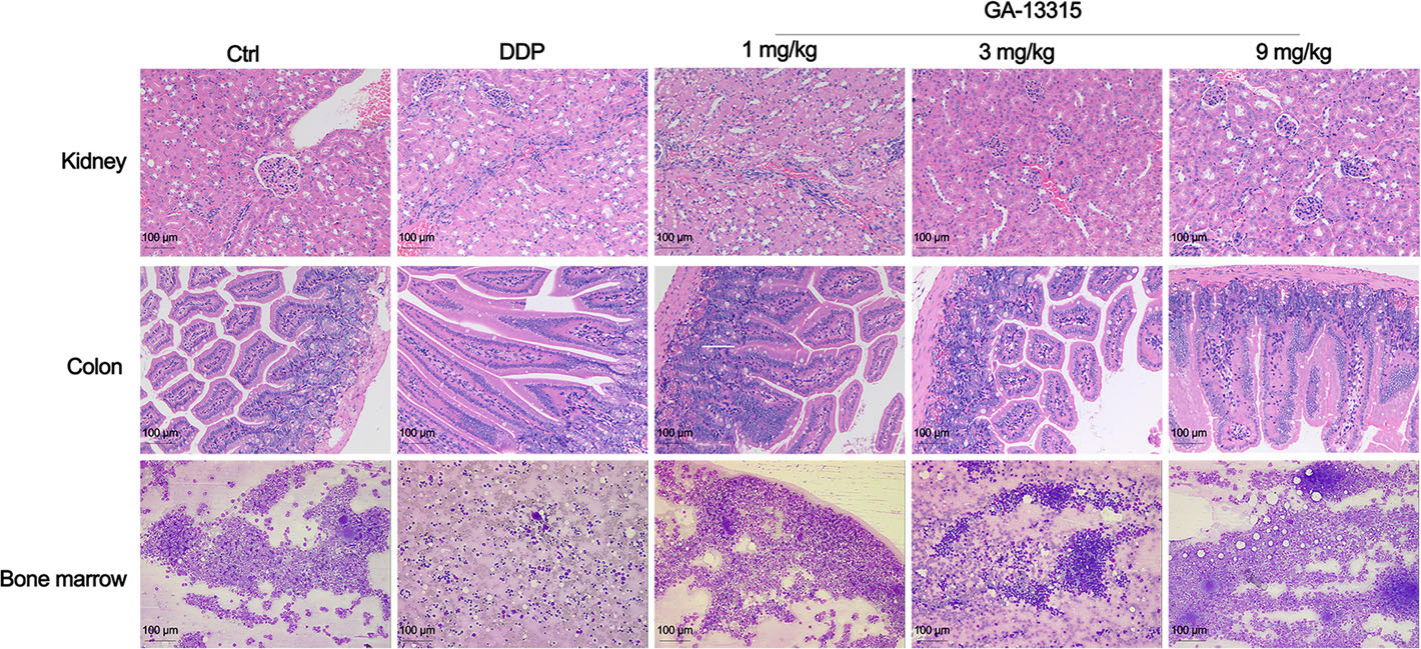
Histopathological examination of bone marrow, kidney and colon tissues in xenograft model of A549 cells in control group and GA-13315 treated groups. Ctrl, control group; DDP, cisplatin

The bone marrow, colon and kidney tissues were collected from mice for histopathological examination. As shown in Fig. 2, GA-13315 treatment groups showed excessive proliferation of bone marrow, and normal granulocytes, lymphocytes, megakaryocytes and platelets were also observed. There was no obvious abnormal colonic structure in the groups. The analysis of the sections of the kidney revealed local kidney interstitial fibrosis in the low and medium dose GA-13315 treated mice, as well as extra focal lymphocytic infiltration in the high dose treatment group. The kidney damage in the DDP group was more severe due to partial glomerular atrophy (30–40%) and local kidney interstitial fibrosis (5–10%).

### GA-13315 triggered apoptosis in A549 cells

**Fig. 3:**
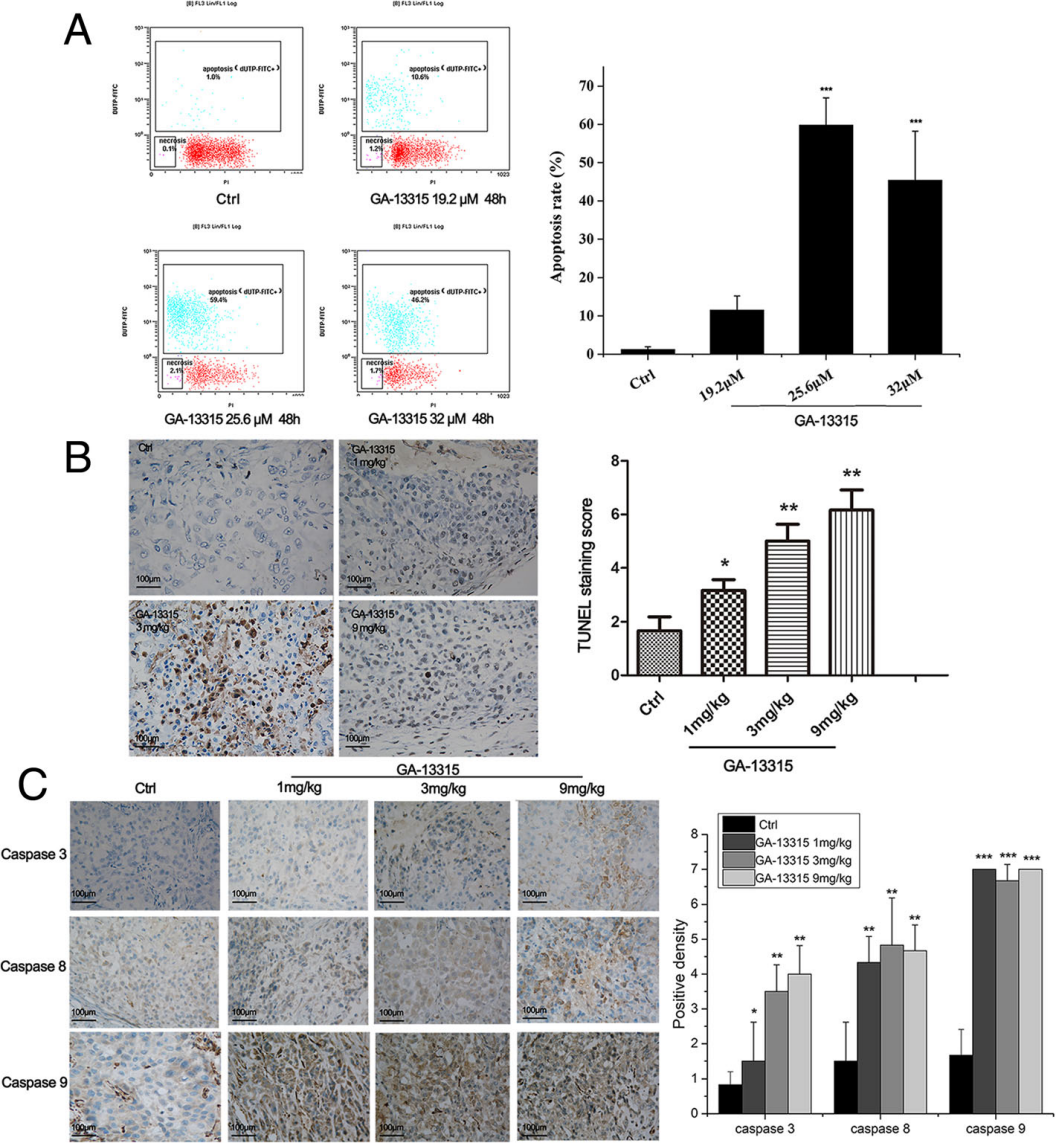
GA-13315 triggered apoptosis in A549 cell line. **a**, Apoptosis was detected in A549 cells by flow cytometry in control group and GA-13315 treated groups; **b**, Apoptosis in tumor tissues was detected by TUNEL staining in control group and GA-13315 treated groups; **c**, Expression of caspase-3, − 8, and − 9 was determined by immunohistochemical analysis in control group and GA-13315 treated groups. Ctrl, control group; ***P < 0.001, compared with control group

According to the results of the preliminary experiments, 19.2 μM, 25.6 μM and 32 μM GA-13315 were administered in A549 cells. After 48 h treatment, flow cytometry was performed to detect cell apoptosis (Fig. 3A). As shown in Fig. 3A, GA-13315 triggered remarkable apoptosis in A549 cells at the concentration of 25.6 μM and 32 μM.

Apoptosis in tumor tissues was also detected by in situ TUNEL staining (Fig. 3B). The nuclei stained brown were considered as positive cells, while rare TUNEL-positive nuclei were found in the control group. The percentage of TUNEL-positive nuclei was significantly increased in GA-13315 treatment groups, particularly in high and medium concentration groups.

The histological characteristics of caspase-3, − 8 and − 9 were further assessed by IHC analysis. Caspase-3, − 8 and − 9 were located in the cytoplasm, and stained brown cells were considered positive cells. Fewer positive cells were observed in the control group. The number of caspase-3 positive cells was significantly higher in the high and medium concentration treatment groups than that in the control group. For caspase-8 and caspase-9, higher positive cell expression was found in three different concentrations of GA-13315 (Fig. 3C).

### Mitochondrial apoptosis pathway in GA-13315 treated cells

**Fig. 4:**
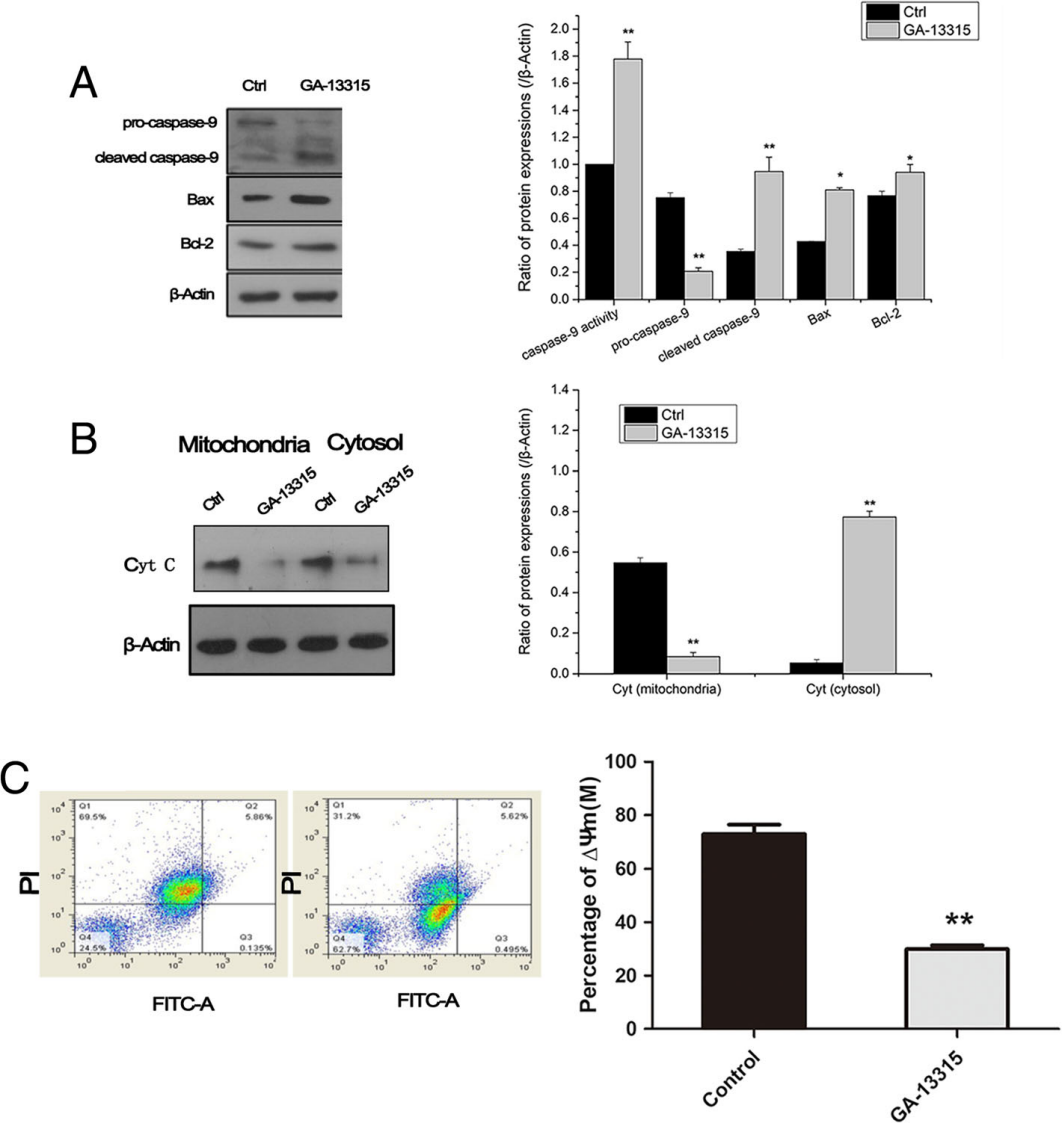
Effect of GA-13315 on mitochondrial apoptosis pathway. **a**, Western blotting for measuring protein expression levels of pro-caspase-9, cleaved caspase-9, Bax and Bcl-2; **b**, Western blotting for detecting protein levels of cytochrome c in mitochondria and cytosol; **c**, Flow cytometry assay for determining mitochondrial membrane potential in control and GA-13315 group. Ctrl, control group; Cyt c, cytochrome c. *P < 0.05, compared with control group; **P < 0.01, compared with control group

To explore whether the mitochondrial pathway was involved in apoptosis induced by GA-13315, we performed subcellular fractionation in A549 cells treated with GA-13315. Increased caspase-9 and cleaved-caspase-9 activities were observed, and the expression levels of Bax and Bcl-2 were significantly elevated in the GA-13315 treated group (Fig. 4A). In addition, the ratio of Bax to Bcl-2 in the GA-13315 treated group was elevated compared with that in the control group. Moreover, the expression of cytochrome c in mitochondria was reduced, while the level of cytochrome c in the cytoplasm was increased, and the differences were statistically significant (Fig. 4B). The MMP in A549 was decreased after GA-13315 treatment (Fig. 4C).

### Endoplasmic reticulum apoptosis pathway in GA-13315 treated cells

**Fig. 5:**
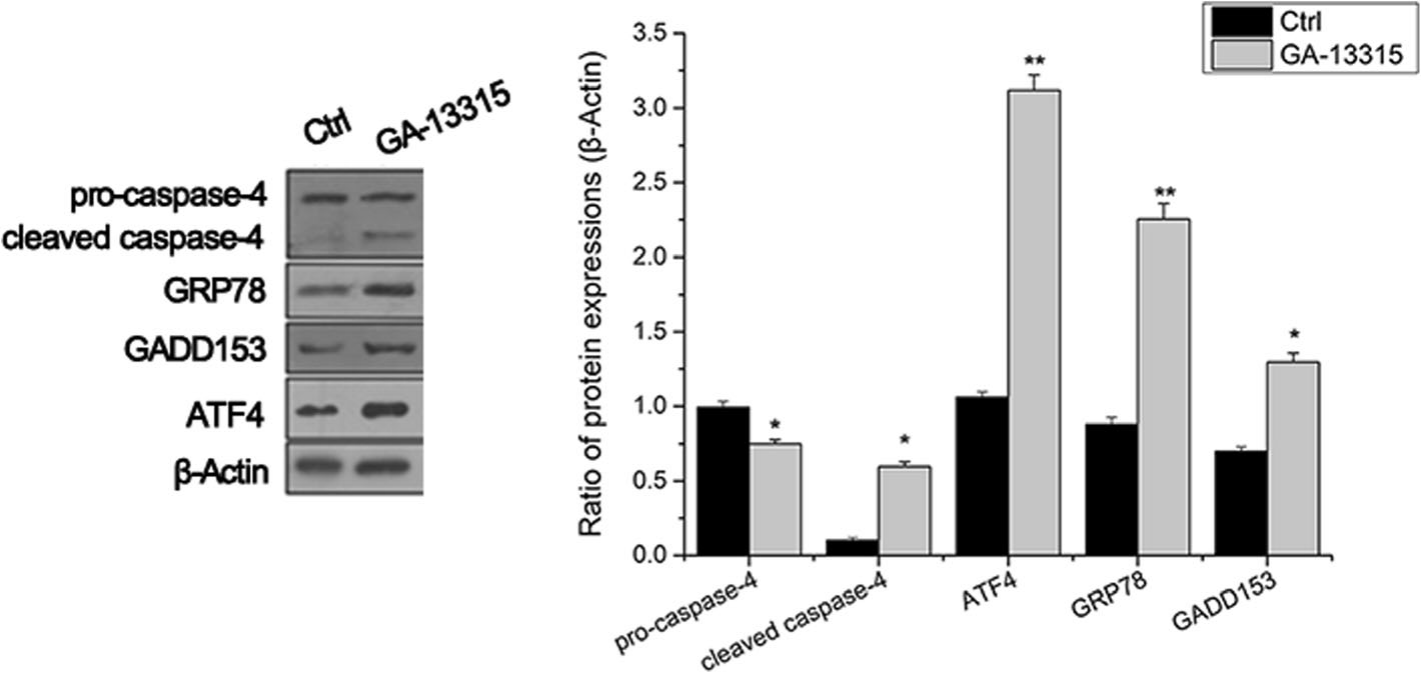
Effect of GA-13315 on endoplasmic reticulum pathway in A549 cells. Western blotting for detecting protein levels of pro-caspase 4, cleaved-caspase 4, ATF4, GRP78 and GADD153 and quantifications analysis of pro-caspase 4, cleaved caspase 4, ATF4, GRP78 and GADD153 levels. Ctrl, control group. *P < 0.05, compared with control group; **P < 0.01, compared with control group

Furthermore, biomarkers of the endoplasmic reticulum apoptosis pathway were also determined. As shown in Fig. 5, protein levels of pro-caspase-4 and cleaved caspase-4 in A549 cells were significantly elevated after GA-13315 treatment. The expression levels of caspase-4, ATF4, GRP78 and GADD153 were both markedly upregulated in the endoplasmic reticulum pathway.

## Discussion

GA-13315, a chemical compound that includes α,β-unsaturated ketone, was proved to enhance the sensitivity of multidrug-resistant cells partially through inhibiting the efflux function of P-glycoprotein in breast carcinoma cells [15]. Our previous study indicated that the antiangiogenic activity of GA-13315 contributed to its anticancer properties [12]; however, the anti-tumor effect of GA-13315 and its mechanism are still poorly elucidated. Therefore, we attempted to explore the role and underlying mechanism of GA-13315 against tumor. In the present study, we found that GA-13315 exhibited potent dose- and time-dependent anti-proliferative activity with low toxicity. Additionally, we provided evidence that GA-13315 promoted apoptosis of A549 cells in vivo and in vitro, and importantly, mitochondria and endoplasmic reticulum signaling pathways were confirmed to be involved in its action.

Bax and Bcl-2 are members of the apoptotic regulator family. Bax has an important pro-apoptotic role in cells, while Bcl-2 is a key protein against apoptosis. Previous studies have shown that an increase in the ratio of Bax to Bcl-2 implies amplification of pro-apoptotic signals or a reduction in anti-apoptotic function [16, 17]. In the present study, the ratio of Bax to Bcl-2 in the GA-13315 treated group was elevated compared with the control group, suggesting an increased tendency of cells to apoptosis.

Furthermore, Bax could induce the permeability of mitochondrial membrane, and lead to the release of pro-apoptotic proteins such as cytochrome c, which eventually caused activation of caspase and cell destruction. Sharifi S [18] revealed that doxorubicin promoted mitochondrial-dependent apoptosis through up-regulating the expression levels of Bax, caspase-8 and caspase-9 in breast cancer cells. Tubeimoside-1 was confirmed to induce apoptosis by increasing the concentration of reactive oxygen species via releasing cytochrome c and activating caspase-3 [19]. Consistent with these experimental results, we found that cytochrome c was released from mitochondria to cytosol, and the MMP was reduced in the GA-13315 group. Additionally, the elevated pro-caspase-9 indicated activation of caspase-9. Our findings suggested that GA-13315 triggered the mitochondria-dependent apoptosis pathway by releasing cytochrome c and activating caspase-9.

The ability of the endoplasmic reticulum (ER) to respond to interference is a fundamental attribute of all cells, but ER stress can also lead to apoptosis. To counteract the pernicious effects of ER stress, three ER stress receptors, pancreatic ER kinase (PKR)-like ER kinase (PERK), inositol requiring enzyme 1 (IRE1) and activating transcription factor 6 (ATF6), which bind to GRP78, mediated the unfolded protein response. On accumulation of unfolded proteins, GRP78 dissociated from the three receptors, thereby resulting in their activation and stress response [20]. In the current study, the expression of GADD153 was markedly upregulated in the GA-13315 treated group, indicating that GA-13315 triggered apoptosis of A549 cells through the endoplasmic reticulum pathway. We analyzed the expression of ATF4, which has been implicated in mediating ER stress-induced apoptosis. The data showed that GA-13315 significantly activated ATF4, implying that the ATF4 pathway was one of the major pathways involved in GA-13315-induced ER stress in the A549 cell line. Our results are in conformity with the previous study. Hongbiao Huang [21] demonstrated that anacardic acid, a natural compound isolated from the traditional medicine *Amphipterygium adstringens*, induced hepatoma HepG2 and myeloma U266 tumor cells’ apoptosis via ATF4-dependent ER stress. GRP78, another marker of ER stress, was identified to be increased after cisplatin challenge [22]. Consistent with this finding, the expression of GRP78 was upregulated in the GA-13315 group in the current study. This implied that GA-13315 induced cell apoptosis associated with induction of the GRP78-dependent endoplasmic reticulum apoptosis pathway.

There are some limitations to the present study. Although we revealed that GA-13315 promoted apoptosis of the A549 cell line via the mitochondria apoptosis pathway and endoplasmic reticulum pathway, other lung adenocarcinoma cell lines should be further investigated. Furthermore, how exactly GA-13315 specifically induced apoptosis through the mitochondrial and endoplasmic reticulum pathways should be addressed in the future.

In conclusion, GA-13315 is confirmed to have low toxicity and anti-cancer efficacy in A549 cells. Additionally, the compound GA-13315 promotes apoptosis of A549 cells through the mitochondria apoptosis pathway and endoplasmic reticulum apoptosis pathway. Our findings might provide new convincing evidence on the development of a chemotherapeutic antitumor drug for patients with lung adenocarcinoma.

## Abbreviations

ATF4: activating transcription factor 4
Bax: Bcl-2-associated X protein
Bcl-2: B-cell lymphoma-2
DDP: cis-dichlorodiamine platinum (II)
EGFR-TKIs: epidermal growth factor receptor -tyrosine kinase inhibitors
GADD153: growth arrest and DNA damage-inducible gene 153
GRP78: glucose-regulated protein 78
IC50: half maximal inhibitory concentration
MMP: mitochondrial membrane potential
MTT: 3-[4,5-dimethylthiazol-2-yl]-2,5 diphenyl tetrazolium bromide)
NSCLC: non-small cell lung carcinoma
T/C%: tumor relative growth rate
